# Spatiotemporal Evolution of Winter Wheat Planting Area and Meteorology-Driven Effects on Yield under Climate Change in Henan Province of China

**DOI:** 10.3390/plants13152109

**Published:** 2024-07-30

**Authors:** Donglin Wang, Mengjing Guo, Shaobo Liu, Yi Li, Qinge Dong, Xuewen Gong, Jiankun Ge, Feng Wu, Hao Feng

**Affiliations:** 1 College of Water Conservancy, North China University of Water Resources and Electric Power, Zhengzhou 450046, China; wangdonglin@ncwu.edu.cn (D.W.); guomengjing0417@163.com (M.G.); gongxuewen@ncwu.edu.cn (X.G.); gejiankun@ncwu.edu.cn (J.G.); wufeng@ncwu.edu.cn (F.W.); 2 School of Water Resources and Environment Engineering, Nanyang Normal University, Nanyang 473061, China; 3 State Key Laboratory of Soil Erosion and Dryland Farming on the Loess Plateau, Institute of Soil and Water Conservation, Northwest A&F University, Yangling 712100, China; liyi@nwafu.edu.cn (Y.L.); nercwsi@vip.sina.com (H.F.); 4 Henan Key Laboratory of Water-Saving Agriculture, Zhengzhou 450046, China

**Keywords:** climate change, winter wheat, planting area, yield, correlation analysis, ArcGIS

## Abstract

This study examines the impact of climate change on winter wheat production in Henan Province. The analysis, under the utilization of GLASS LAI data, focuses on shifts in the planting areas of winter wheat. In addition, a comprehensive assessment of the spatiotemporal trends in meteorological factors during the winter wheat growth period has also been conducted. The findings reveal a fluctuating increase in accumulated temperature across Henan Province, ranging from 3145 °C to 3424 °C and exhibiting a gradual rise from north to south. In particular, precipitation patterns from 1980 to 2019 showed limited significant trends, while notable abrupt changes were observed in 1983, 2004, 2009, and 2016. Geographically, southwestern Henan Province experiences greater precipitation than the northeast. Moreover, a fluctuating downward trend in sunshine hours has been observed, gradually decreasing from north to south. The study further highlights an increase in winter wheat planting frequency in the northwestern region of Luoyang and the northeastern part of Zhumadian, contrasted by a decrease in Zhengzhou and Kaifeng. Accumulated temperature is positively correlated with the expansion of winter wheat planting areas (R^2^ = 0.685), while sunshine hours exert a suppressive effect (R^2^ = 0.637). Among meteorological factors, accumulated temperature emerges as the most crucial determinant, followed by precipitation, with sunshine hours having a relatively minor influence. Yield demonstrates a positive association with accumulated temperature (R^2^ = 0.765) and a negative correlation with sunshine hours (R^2^ = −0.614). This finding is consistent with the impact of meteorological factors on winter wheat production. The results of this study enhance the understanding of how the underlying mechanisms of climate change impact crop yields.

## 1. Introduction

Wheat, renowned for its remarkable adaptability, is cultivated extensively across the globe, occupying vast tracts of agricultural land [1,2,3]. It serves as a pivotal protein source for numerous developing nations and holds a preeminent position as a protein crop in terms of cultivated area [4,5]. Notably, China excels in wheat production and consumption, accounting for 17% of the global wheat output and contributing 22% to the country’s overall grain production [6,7,8], with the largest traditional planting area [9]. Therefore, it is of great significance to obtain accurate winter wheat planting area information and its spatial distribution in time, so as to fulfil its production potential, which will promote agricultural production development and food security in China [10]. The two main methods to obtain the information of the winter wheat planting area are statistical data and remote sensing monitoring [11,12], among them, multitemporal remote sensing data classification technology is one of the effective methods to obtain the spatial distribution and quantity of planting area with high accuracy and full coverage [12,13].

Nestled in the heart of the Yellow and Huaihai Plains, Henan Province boasts abundant light and heat resources, positioning it as one of China’s most developed agricultural regions [14,15,16]. Henan plays a vital role in ensuring national food security, with winter wheat cultivation comprising approximately 25% of the country’s total wheat area and contributing roughly 24% to national wheat output [15,17]. Furthermore, Henan annually supplies 25–30% of the nation’s commercial wheat, maintaining its position as a leading wheat producer nationwide [18]. Deng et al. (2019) used time-series MODIS remote sensing images and determined threshold values with the help of statistical data to extract the winter wheat area and its spatiotemporal variation in Henan Province from 2004 to 2013 [19]. Ning et al. (2016) used NDVI remote sensing images in a time series and a support vector machine algorithm to extract the winter wheat planting area of the semiarid hilly areas of Shanxi Province [20]. The above research proves that the method of extracting winter wheat planting area using remote sensing is a feasible method, and the spatiotemporal distribution of winter wheat area in Henan Province is relatively stable. However, winter wheat is highly sensitive to weather conditions. Woli evaluated the influence of the El Niño-Southern Oscillation (ENSO) on the crop yields in the Llano Estacado region of the southern United States using the DSSAT crop model and 81 years of weather data [21]. More and more studies have begun to focus on how climate change affects wheat yields and the development trend of winter wheat planting area. Li et al. (2021) combined a cropping system model (CropSyst) with a geographic information system (GIS) to build a crop yield simulation model [22]. Ding et al. (2022) used the geodetector model to explore the influence of various meteorology-driven effects, such as precipitation and temperature, on the NDVI changes in the study area [23].

Currently, the primary wheat production regions in China are predominantly situated in the Yellow Huanghuaihai region, the middle and lower sections of the Yangtze River, and the southwestern region [24,25,26]. The Yellow Huaihai region encompasses provinces such as Henan, Shandong, Hebei, Jiangsu, and Anhui. As the largest wheat-producing area in China, the Yellow Huaihai region contributes over 40% to the national wheat output [27,28,29]. Research in this area has been limited in terms of examining the interrelation between meteorological variables and wheat cultivation. Furthermore, studies evaluating the predictive modeling of winter wheat yield in Henan Province under varying precipitation scenarios are scarce. In addition, investigations that encompass the entire province and delve into the interplay between climate elements and wheat cultivation area and yield, as well as the trends in planted area and climate yield, are notably lacking. Given that Henan Province is the key producer of winter wheat in China, this study aims to conduct a comprehensive analysis of the specific impact of climate change on the planting area and winter wheat yield.

In this paper, the GLASS LAI data are used to employ the inflection point method and threshold method for precise identification of key inflection points and peaks on the LAI characteristic curve. These are the fundamental parameters in the remote sensing extraction process. Subsequently, dryland data from remote sensing monitoring in China are utilized to extract information on dryland crops, particularly wheat. Crop planting grid points have been determined based on the establishment of three distinct climatic periods for each crop to ensure their simultaneous recognition [19,30]. To enhance data accuracy, the LAI maximum threshold method has been employed to eliminate misidentified crop planting grid points. These procedures provide great facilitation to the acquisition of winter wheat distribution data in the study area from 2000 to 2019, enabling further analysis of winter wheat planting area trends. In addition, meteorological observations are also involved in this study to investigate the spatial and temporal trends of meteorological factors during the winter wheat growth period. On the basis of detailed correlation analysis and importance assessment, the specific impact of climatic-factor changes on winter wheat planting area and yield has been comprehensively examined to provide a valid results assessment. In general, this study not only enhances the understanding of the impact of climate change on crop production but also provides a crucial scientific foundation for future agricultural practices and climate adaptation strategies.

## 2. Results

### 2.1. Spatial and Temporal Distribution Characteristics of Meteorological Factors

#### 2.1.1. Temporal Distribution Characteristics

**Figure 1:**
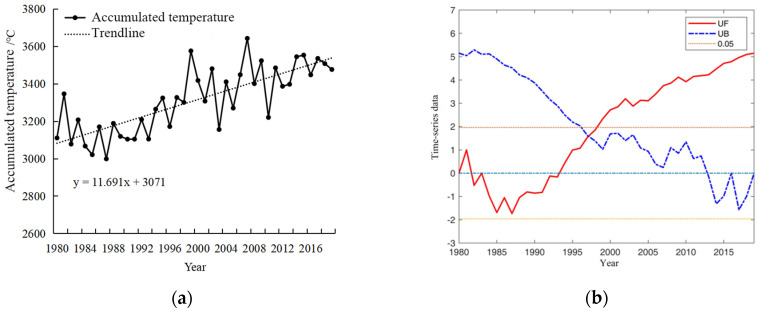
Trend changes in the mean accumulated temperature during the winter wheat growth period in Henan Province, 1980–2019 (**a**) and mutability test plots (**b**).

The spatial and temporal distribution characteristics of precipitation, accumulated temperature, and sunshine duration during the growth period of winter wheat in Henan Province were analyzed in detail, which prepared the data for further study on the meteorology-driven effects on the planting area and yield of winter wheat. Accumulated temperature is an important caloric index to measure agricultural climate. The greater the accumulated temperature, the more it can meet the crop growth requirement. Figure 1a depicts the pattern of accumulated temperature observed during the winter wheat growth period in Henan Province from 1980 to 2019. The mean accumulated temperature recorded stands at 3310.71 °C, exhibiting a fluctuating trend with an average increase of 116.91 °C per decade, displaying fluctuating trends. The variability in accumulated temperature during the winter wheat growth period in Henan Province from 1980 to 2019 is further analyzed and presented in Figure 1b. The UF statistical curve derived from the Mann–Kendall test method exhibited fluctuations within the critical range (between two significant test lines and within the confidence interval) from 1980 to 1998, indicating that the trend and variability in accumulated temperature were not distinctly pronounced during this period. Notably, between 1982 and 1993, the UF value remained below zero, suggesting a gradual decline in accumulated temperature. Conversely, after 1998, the UF curve predominantly remained above zero, indicating a gradual increase in accumulated temperature. The intersection of the UF curve and the UB curve in 1997 signifies a sudden shift in the accumulated temperature around that year.

**Figure 2:**
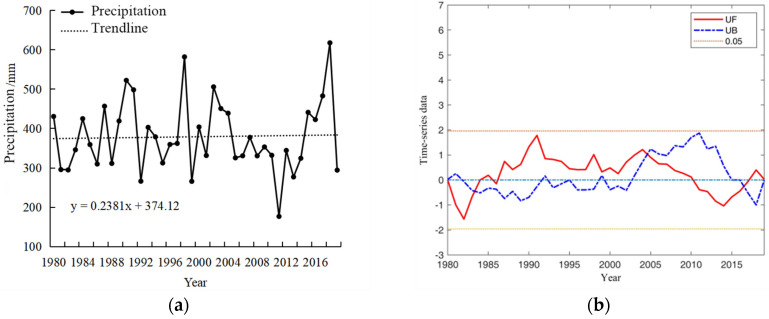
Changes in precipitation trends (**a**) and mutability test plots (**b**) during the winter wheat growth period of winter wheat in Henan Province, 1980–2019.

Figure 2a shows the variability in precipitation patterns observed during the winter wheat growth period from 1980 to 2019. The precipitation trends from 1980 to 2019 remain relatively stable, with notable changes occurring in 1983, 2004, 2009, and 2016. The average annual precipitation recorded was 379 mm, peaking at 617.65 mm in 2018. Figure 2b provides an analysis of precipitation variability during the reproductive phase of winter wheat between 1980 and 2019. The UF curve predominantly remained within the critical threshold, indicating that there was neither a significant discernible trend nor substantial fluctuations in precipitation levels during the reproductive phase. Notably, mutation points in the average precipitation were identified approximately in the years 1983, 2004, 2009, and 2016.

**Figure 3:**
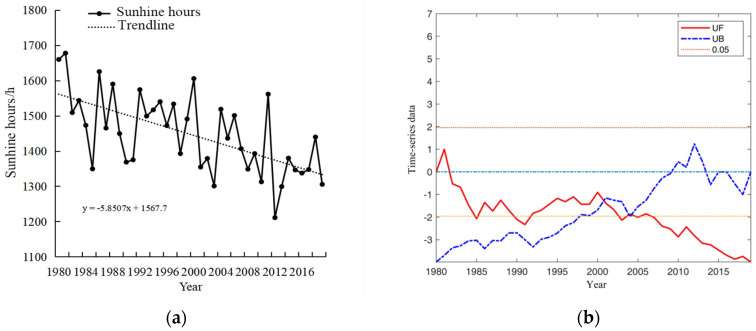
Trend change in sunshine hours (**a**) and mutability test plots (**b**) during the winter wheat growth period in Henan Province, 1980–2019.

Figure 3a shows the evolution of sunshine hours during the winter wheat growth period in Henan Province from 1980 to 2019. The annual average sunshine hours were recorded at 1447.8 h, exhibiting a gradual decline with fluctuations. Figure 3b shows the variability in average sunshine hours during the same period in Henan Province from 1980 to 2019. The intersection points of UF and UB in 2001, 2003, and 2005 signify a mutation in the sunshine hours during these specific years. Apparently, a significant decrease in sunshine hours was observed after 2007.

#### 2.1.2. Spatial Distribution Characteristics

**Figure 4:**
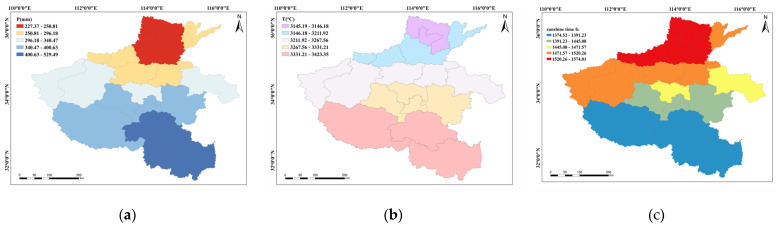
Spatial distribution of the mean values of precipitation (**a**), accumulated temperature (**b**) and the sunshine hours (**c**) during the winter wheat growth period in Henan Province, 1980–2019.

The precipitation trend in Henan Province (Figure 4a) exhibits a remarkable disparity, with significantly higher levels observed in the southern and southeastern regions compared to the northern and northwestern areas. Specifically, the capital city of Zhengzhou experienced substantial seasonal variations in precipitation levels, with the highest amount occurring during summer, accounting for 60% of the annual total. Correspondingly, the lowest amount occurred in winter, comprising less than 5% of the yearly precipitation. The topography of the region, particularly the Funiu and Dabie mountains, has a profound impact on precipitation distribution, resulting in mountainous areas receiving approximately twice as much rainfall as the plains. Furthermore, disparities in precipitation levels across different regions within Henan Province were self-evident, with mountainous regions in the south receiving significantly more precipitation than the northern plains. The spatial distribution of precipitation in Henan Province is intricate and influenced by diverse natural factors. What is more, the accumulated temperature throughout the whole winter wheat growth period (Figure 4b) in Henan Province ranges from 3145 °C to 3423 °C, exhibiting a clear trend of higher temperatures in the southern areas and lower temperatures in the north. In addition, eastern regions experience higher temperatures, while the western regions are relatively cooler. Furthermore, significant temperature variations are observed between mountainous and plain areas, accompanied by substantial interannual and diurnal temperature fluctuations. In addition to that, the sunshine hours during the winter wheat growth period in Henan Province (Figure 4c) range from 1374 to 1574 h, with a trend of more sunshine hours in the northern plains compared to the southern regions and more sunshine hours in the plains than in mountainous areas. In recent years, Henan Province has experienced a rise in air pollution levels, resulting in a decrease in wind speed, the accumulation of atmospheric aerosols at lower altitudes, an increased air humidity, and a higher frequency of light-fog days. These phenomena have collectively led to a reduction in atmospheric transparency and sunshine duration, significantly impacting the spatial distribution characteristics of sunshine hours in the region.

### 2.2. Changes in the Planting Area of Winter Wheat

#### 2.2.1. Evaluation of the Extraction Accuracy of Winter Wheat

**Figure 5:**
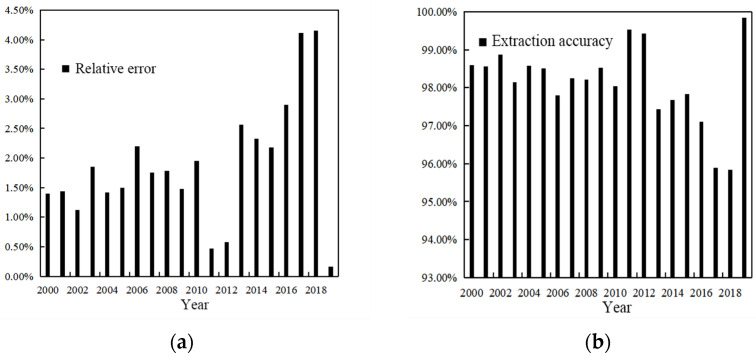
Relative error (**a**) and extraction accuracy (**b**) of the remote-sensing-extracted planting area and statistical planting area of winter wheat in Henan Province.

Figure 5 presents a comparative analysis between the relative error and extraction accuracy of the planting area of winter wheat in Henan Province, derived through remote sensing techniques, and the corresponding statistically determined planting area. The findings reveals that the relative errors associated with the extraction of spatial distribution information for winter wheat did not exceed 4.16%. Moreover, the precision levels of the sample points varied from 95.84% to 99.84%, averaging 98.13%. These results indicate a remarkably high level of accuracy in the extraction of the spatial distribution of winter wheat.

#### 2.2.2. Extracted Results for the Planting Area of Winter Wheat

**Figure 6:**
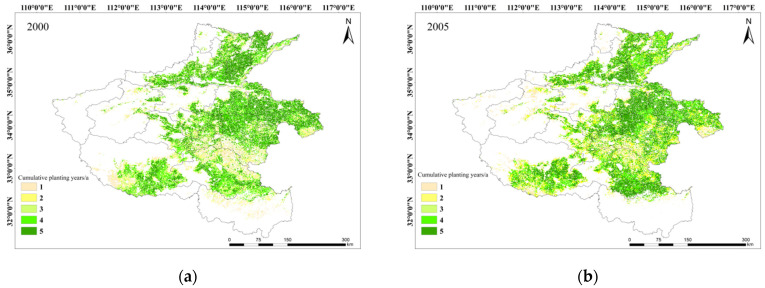

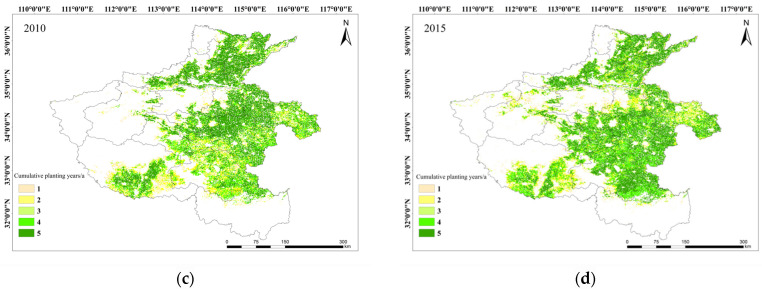
Distribution of the planting area of winter wheat during 2000–2004 (**a**), 2005–2009 (**b**), 2010–2014 (**c**) and 2015–2019 (**d**) in Henan Province.

Figure 6 illustrates the spatial distribution of winter wheat cultivation in Henan Province spanning from 2000 to 2019. The findings can be highlighted in a better way in five-year intervals, since the spatial variability in winter wheat cultivation across years has been proven to be insignificant. The figure reveals that winter wheat cultivation in Henan Province is widespread, primarily concentrated in the central, eastern, and northern regions, encompassing key agricultural regions such as Zhoukou, Shangqiu, and Xinxiang. Conversely, regions like Nanyang in the southwest and the mountainous city of Sanmenxia exhibit relatively smaller planting areas for wheat cultivation. This is primarily due to the prevalence of mountainous and hilly terrain, though such topographies have the potential for winter wheat growth. The successful growth of winter wheat is contingent upon specific climatic conditions, including adequate precipitation, suitable temperatures, and ample sunlight. The failure to meet these conditions might impede the planting and growth of wheat to some extent. Over the years of continuous monitoring, the spatial distribution of winter wheat cultivation displayed no discernible trend of change, maintaining a relatively stable overall layout. Nevertheless, a comparative analysis of planting patterns across different regions reveals that the central–eastern region exhibits a higher density of wheat cultivation compared to the southwestern region, attributable to factors such as topography and soil quality.

#### 2.2.3. Spatial Distribution of Winter Wheat Planting Frequency

**Figure 7:**
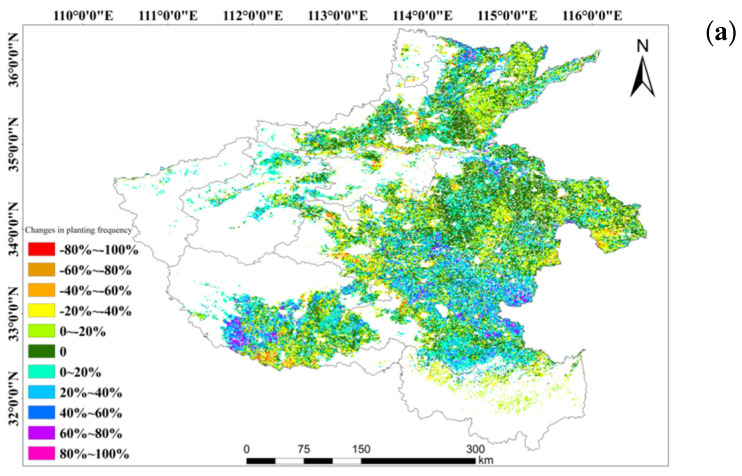

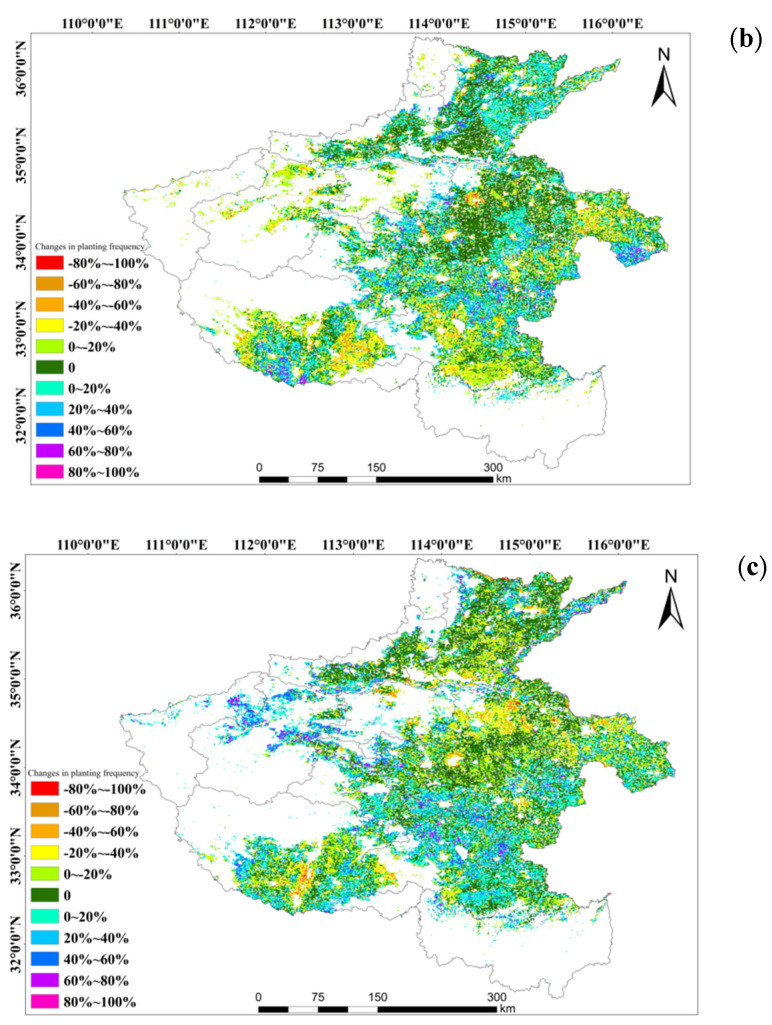
Changes in the frequency of winter wheat cultivation within each interval as compared between 2000~2005 and 2005~2010 (**a**), 2005~2010 and 2010~2015 (**b**), and 2010~2015 and 2015~2019 (**c**), respectively.

The time span from 2000 to 2019 was segmented into four equal intervals, and the data were analyzed individually to assess the changes in winter wheat cultivation in Henan Province. As shown in Figure 7, a value of 1 was assigned to regions where winter wheat is planted and regions with a value of 0 represent regions with no crops. The frequency of planting was finally determined after the examination of winter wheat distributions across the intervals. The quantitative analysis reveals that the primary regions for winter wheat cultivation in Henan Province from 2000 to 2019 are the central, northern, and eastern parts, as illustrated in Figure 7. Spatial variations are observed in winter wheat planting frequency changes. In particular, regions with stable planting frequencies are predominantly situated in the east–central area, including the southeastern regions of Anyang, Xuchang, Zhoukou, and Luohe. On the other hand, areas with significant fluctuations in planting frequency are concentrated in the southern part of Nanyang City, the western part of Zhumadian City, and the southwestern part of Kaifeng City. The frequency of winter wheat planting notably increases from the first to the second interval in Henan Province, with a larger expansion in regions experiencing an increase compared to those with a decrease in planting frequency. Subsequently, from the second to the third interval, there is a general increase in planting frequency, with growth concentrated in the northeastern region and reductions in the southwestern and central regions. Finally, an overall rise in winter wheat planting frequency can be seen from the third to the fourth interval, and this is particularly true for the northwestern region of Luoyang, the northern of Pingdingshan, and the northeastern of Zhumadian.

### 2.3. Analysis of the Effect of Climatic Factors on the Planting Area and Yield of Winter Wheat

**Figure 8:**
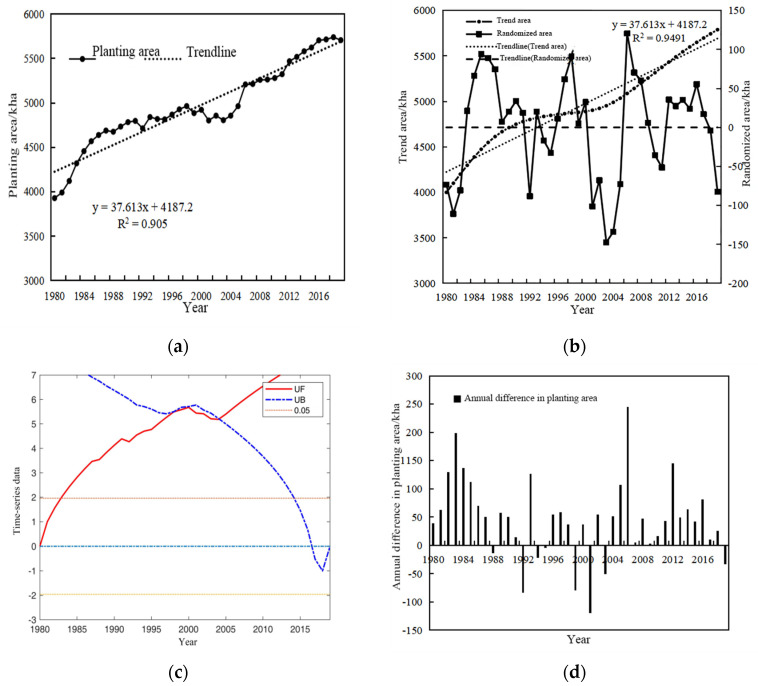
Plots of actual planting area (**a**), trend area (**b**), test of mutability of actual planting area (**c**) and annual difference in planting area (**d**) for winter wheat in Henan Province, 1980–2019.

**Figure 9:**
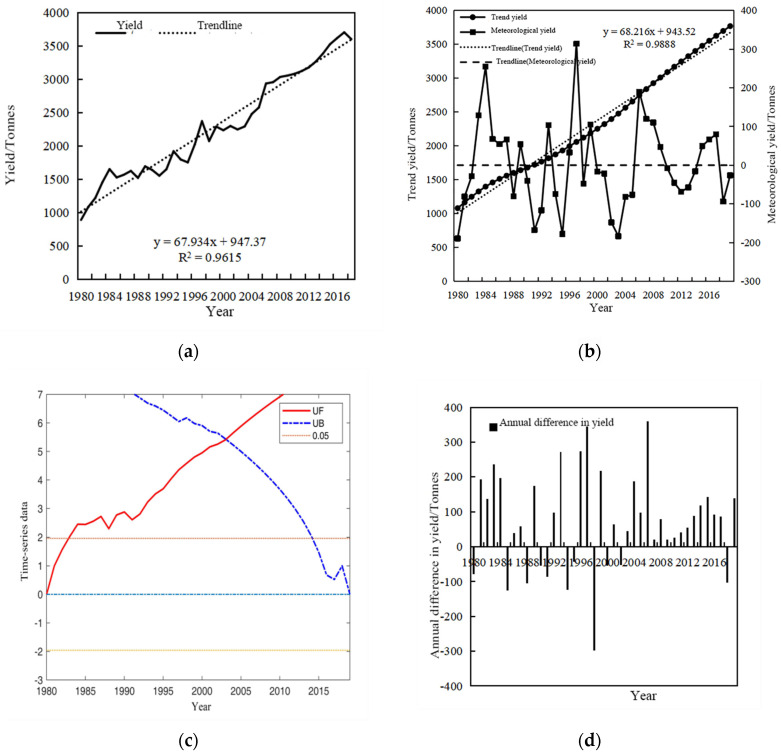
Winter wheat yield map (**a**), trend yield map (**b**), yield mutability test map (**c**) and yield yearly difference map (**d**) in Henan Province, 1980–2019.

Figure 8a,b shows the recorded and projected trends in the planting area of winter wheat in Henan Province spanning from 1980 to 2019. A comprehensive analysis of the data reveals a consistent upward trajectory in both the actual and projected planting areas during this period. Specifically, Figure 8a shows that the lowest point of the recorded planting area observed in 1980 is a mere 3927 k ha. This initial datum provides a baseline for understanding the subsequent growth and expansion in winter wheat cultivation in the province. Figure 8b, on the other hand, portrays the projected trajectory of winter wheat cultivation, mirroring the upward pattern observed in the actual planting area. This projected growth highlights the enduring significance and expanding scope of winter wheat cultivation in Henan Province. Figure 8c incorporates the UF (Mann–Kendall trend test) curve for the actual planting area from 1980 to 2019 so the variability in winter wheat cultivation can be further examined. Notably, the UF curve remained within the critical threshold from 1980 to 1983, indicating a period of stagnation in the planting area. However, subsequent to 1983, the UF curve surpassed the critical threshold, signifying a notable and sustained increase in the actual planting area of winter wheat. This upward shift in the UF curve serves as a compelling indicator of the growing prominence and cultivation of winter wheat in Henan Province. Figure 8d presents the annual fluctuations in winter wheat sowing in Henan Province over the 1980–2019 period. A notable peak is observed in 2006, when the sowing area reached 245.77 k ha. This significant peak underscores a particularly prosperous year for winter wheat cultivation in Henan Province, likely influenced by favorable weather conditions, advancements in agricultural technology, or other factors conducive to crop growth and yield. In general, Figure 9 presents a rigorous and comprehensive analysis of the recorded and anticipated trends in winter wheat cultivation in Henan Province, revealing both the upward trajectory and annual variations in this important agricultural commodity.

The actual and trend yields of winter wheat in Henan Province from 1980 to 2019 are presented in Figure 9a,b. It is evident that both the actual and trend winter wheat yields exhibit an overall upward trend during this period. The mutability test of actual winter wheat yield is shown in Figure 9c. Initially, between 1980 and 1983, the UF curve remains within the critical line, suggesting the absence of a distinct growth trend in the actual yield during this period. However, after 1983, the UF curve surpasses the critical line, coinciding with a significant increase in the actual planting area. The annual difference in the actual sowing of winter wheat in Henan Province from 1980 to 2019 is presented in Figure 9d. It is apparent that there was a substantial decrease in production in both 1985 and 1994, likely due to unfavorable conditions or other external factors. Conversely, production experienced a remarkable increase in 2006, with an annual difference of up to 3,588,100 tons. This substantial rise highlights a particularly favorable year for winter wheat production in Henan Province.

**Figure 10:**
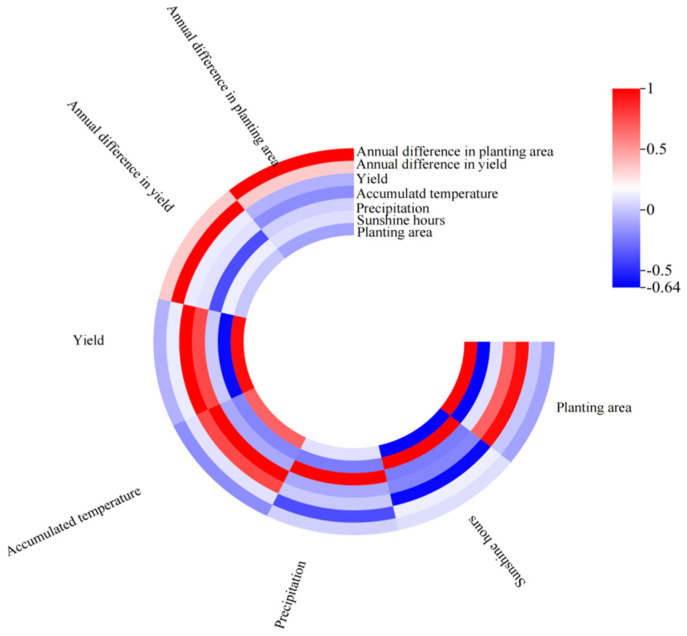
Correlation coefficient graph of meteorological factors (accumulated temperature, precipitation, and sunshine hours) and yield during the winter wheat growth period.

The relationship between meteorological factors (accumulated temperature, precipitation, and sunshine hours) and the planted area of winter wheat during its reproductive phase has been examined and presented in Table 1 and Figure 10. With the help of a Pearson correlation analysis and significance assessment using *p*-values, the study found that the planted area exhibits a positive correlation with accumulated temperature, yielding the strongest correlation coefficient of 0.685. In contrast, a negative correlation was observed with sunshine duration, with a correlation coefficient of −0.637. However, the correlation with precipitation was relatively weak. Further analysis reveals that the variations in annual planted area are negatively associated with both accumulated temperature and sunshine hours. Nonetheless, it is important to note that the statistical significance tests for these associations do not meet the threshold of *p* < 0.05, indicating that the observed effects are not statistically significant. This suggests that while there are discernible trends in the data, the meteorological factors examined in this study do not have a significant, predictable impact on the annual variations in the planted area of winter wheat.

**Figure 11:**
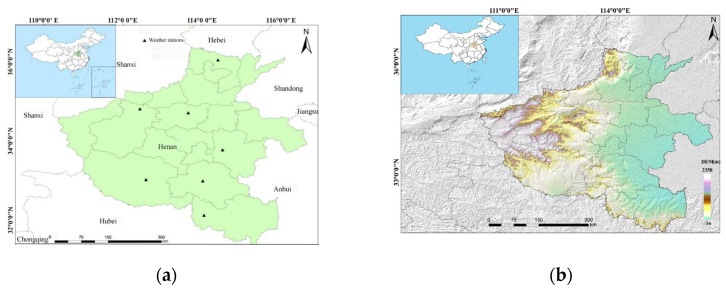
Distribution of the meteorological stations (**a**) and the digital elevation model map (DEM) (**b**) of Henan Province in China.

The correlation coefficients between meteorological factors (including accumulated temperature, precipitation, and sunshine hours) and winter wheat yield during the growth period are presented in Table 1 and Figure 10. The results show that there is a positive and strong correlation between winter wheat yield and accumulated temperature during the growth period, evidenced by a correlation coefficient of 0.765. Conversely, a negative correlation can be observed between winter wheat yield and sunshine hours during the winter wheat growth period, indicating a potentially detrimental impact of extended daylight periods, with a correlation coefficient of −0.614. Furthermore, statistical testing (*p* < 0.05) confirmed a significant negative correlation between the annual variation in yield and precipitation levels, with a correlation coefficient of −0.393. In addition, there was a significant positive correlation between the planting area and winter wheat yield, as shown in Figure 11; with the increase in winter wheat planting area, the yields also increased. This is similar to the conclusion of our previous paper [31].

These findings suggest that an increase in accumulated temperature during the growth period positively affects winter wheat yield, while an increase in sunshine duration during this period negatively impacts yield, both of which effects are statistically significant. These insights can be utilized by farmers and agricultural policy makers to refine crop management strategies and adapt to changing climatic conditions.

The above research indicates that the accumulated temperature and precipitation experienced during the reproductive phase exhibits a positive impact on the planting area of winter wheat. Conversely, the number of sunshine hours somewhat inhibits this planting area. The significance of meteorological factors during the winter wheat growth period in relation to planting area can be prioritized as follows: accumulated temperature during the growth period is of the highest importance, followed by precipitation, and then sunshine hours. Within a specific threshold, increasing accumulated temperature and precipitation levels during this critical growth stage is beneficial for the enhancement of winter wheat yield. Conversely, the duration of sunshine hours during the growth period exhibits a suppressive impact on yield. Consequently, the significance of meteorological factors for winter wheat cultivation in Henan Province, as indicated by this study, can be ranked in the following order: *T* > *P* > *h*.

## 3. Materials and Methods

### 3.1. Description of the Study Area

Henan Province, commonly abbreviated as Yu, is situated in the southern region of the North China Plain, spanning latitudes 31°23′ N to 36°22′ N and longitudes 110°21′ E to 116°39′ E. Covering an extensive area of 167,000 km^2^ [32], Henan Province boasts an annual winter wheat cultivation area exceeding 80 million mu, accounting for a quarter of the national production [33,34,35]. Geographically, it is bordered by Anhui and Shandong to the east, Hebei and Shanxi to the north, Shaanxi to the west, and Hubei to the south.

Climatically, Henan Province experiences a diverse range of patterns, encompassing both temperate monsoon and subtropical monsoon climates. During the summer season, temperatures rise with relatively abundant precipitation, while the winter is characterized by cold and dry conditions. The province enjoys ample heat and light resources, creating optimal conditions for crop cultivation. Figure 11 depicts a map of the study area, highlighting the geographical location and extent of Henan Province.

### 3.2. Data Collection

Winter wheat commonly is planted in the early and middle of October each year and harvested in late May and early June of the next year. According to the phenological characteristics of winter wheat, the remote sensing image data were selected from early October (day 273) to late May (day 145) of the following year. In order to meet the target of remote sensing monitoring in a time series, 145 m by 250 m spatial resolution 16 d synthesis MODISNDVI data (http://modis.gsfc.nasa.gov) were downloaded to further complete the MODIS data processing.

In this study, comprehensive meteorological data spanning from 1980 to 2019 were procured from seven national-level basic meteorological stations in Henan Province, China, through the China Meteorological Data Sharing Network (http://data.cma.gov.cn/). The dataset encompasses the daily records of key meteorological elements, including the maximum and minimum temperatures, precipitation, and sunshine hours. In addition, historical winter wheat yield data were retrieved from the official website of the Henan Provincial Bureau of Statistics (https://www.henan.gov.cn/) and further supplemented by consulting statistical yearbooks from seventeen prefectures and cities, as well as rural statistical yearbooks of Henan. These are actually the most comprehensive collection of winter wheat trial data and yield statistics.

### 3.3. Research Methods

#### 3.3.1. The M-K Trend Test

In this study, the Mann–Kendall (M-K) trend test was employed to analyze potential mutations in the meteorological variables. The M-K test serves as a nonparametric statistical method that is specifically designed to detect significant changes or trends in meteorological data, even when the data do not conform to a normal distribution [36]. The mathematical formula for this test is conventionally expressed as

(1) $$Z=\left\{\begin{array}{cc} \frac{S-1}{\sqrt{Var(S)}} & S>0\\ 0 & S=0\\ \frac{S+1}{\sqrt{Var(S)}} & S<0 \end{array}\right\}$$

where *Z* is a statistical variable, and *S* is the statistic tested by time series *x_i_* with *n* samples in the sequential case; the significance level of this paper is 0.05, and the critical value *Z*_0.05_ = ±1.96.

The *S* formula is

(2) $$S=\sum_{i=2}^{n}\sum_{j=2}^{i=1}sign(X_{i}-X_{j})$$

where the sign function is denoted as sign( ), which can take −1, 0, or 1, *X_i_*, *X_j_* are time-series data, and *n* is the number of samples.

#### 3.3.2. Extraction Method of the Spatial Distribution Information of Winter Wheat

The remote sensing monitoring method of crop area mainly uses the unique spectral reflection characteristics and spatial characteristics of vegetation to separate the crop growing area from the non-growing area. We utilized the globally produced 500 m Terra and Aqua combined Moderate Resolution Imaging Spectroradiometer (MODIS) Land use Type (MCD12Q1) Version 6 dataset spanning from 2000 to 2019 (Wu et al., 2024) and the Nadir Bidirectional Reflectance Distribution Function-Adjusted Reflectance data, with a spatial resolution of 500 m and a daily temporal resolution (16-day composite). All MODIS data were acquired from the National Aeronautics and Space Administration (NASA) (https://search.earthdata.nasa.gov/). Before generating the vegetation index datasets, these data were mosaiced and clipped using Matlab R2023 and ENVI 5.4 software. Furthermore, ArcGIS was used to reproject the data when producing the map; detailed method references can be found in [37,38].

In addition, Jin et al. (2017) reported that the spatial distribution of GLASS LAI products and their statistical relationship with a topographic index, time, and vegetation type were studied, indicating a spatiotemporal consistency [39]. The validation study of leaf area index (LAI) products of GLASS performs similarly to MODIS through comparison with the field LAI data. By applying the inflection point and threshold method to the analysis of GLASS LAI data, we successfully identified the inflection points and peaks that correspond to the leaf area index (LAI) characteristic curve. These identified points represent crucial climatic information obtained through remote sensing techniques. Subsequently, we utilized remote sensing data on China’s current land use status to isolate dryland crops, particularly wheat. By aligning the three crucial climatic periods defined for each crop, we were able to determine optimal planting locations. To enhance the accuracy of our results, we eliminated inaccurately classified crop planting locations through the application of a threshold method based on the maximum LAI. This rigorous process enabled us to generate data on the spatial distribution of winter wheat in the study area spanning from 2000 to 2019 [40].

#### 3.3.3. Accuracy Evaluation

Following the extraction of spatial distribution data pertaining to winter wheat, the planting area of winter wheat was determined. Subsequently, this figure was juxtaposed with the corresponding data on winter wheat planting area as reported in the statistical yearbook, in order to ascertain the relative error between the two. The formula for calculating this relative error is outlined as follows:

(3) $$\delta =\frac{\left|A_{e}-A_{r}\right|}{A_{r}}\times 100\%$$

where *δ* is the relative error, *A_e_* is the area of winter wheat extracted through remote sensing, and *A_r_* is the planting area of winter wheat in the statistical yearbook.

#### 3.3.4. HP Filtering Method

The HP filtering technique, which enjoys widespread use in economics, is designed to eliminate the trend component from a time series, resulting in a significant reduction in variance. Originating from the work of Hodrick and Prescott (1980), this method conceptualizes the time series as a composite of diverse frequency components [41], similar to the operating principle of a high-pass filter. The HP filtering approach segregates the time-series components into high-frequency and low-frequency parts. This segregation enables it to isolate the high-frequency elements with precision while eliminating the low-frequency components effortlessly. This approach facilitates a nuanced examination of the impact of climate change on crop yields. As a result, the HP filtering method is widely acknowledged as a well-established analytical tool in the field of economics [42,43].

## 4. Discussion

### 4.1. Temporal and Spatial Variations of Meteorological Factors during the Winter Wheat Growth Period of Winter Wheat

In recent decades, with the gradual deepening of the impact of climate change, China has also entered a gradual “warming period”. Meanwhile, the main meteorological driving factors, such as precipitation, accumulated temperature, sunshine, etc., have also undergone corresponding changes, and the impact on agriculture has gradually emerged. Therefore, in order to study the impact of climate change on crop production in a local planting region, it is necessary to pay attention to the trend of regional meteorological factors [44]. Wang et al. (2022) reported that the average temperature in China has increased significantly, with obvious stage changes and regional differences, and drought in the North China Plain has become more and more serious, particularly the spring drought which reached 65%, which seriously affected the agricultural ecosystem in the region [9]. Driven by precipitation and temperature, winter wheat yield in the North China Plain has been greatly affected [45]. Feng et al. (2023) reported that the average temperature and total sunshine hours were most sensitive to winter wheat yield in Henan Province during the growing period, followed by precipitation [46]. This is basically consistent with our findings. Our results showed that the accumulated temperature in Henan Province exhibits a fluctuating increasing trend, undergoing a sudden shift around 1997. Geographically, the accumulated temperature varied significantly from north to south, with values ranging from 3145 to 3424 °C, indicating a tendency of higher temperatures in the southern regions and lower temperatures in the northern areas. Notably, there was a distinct temperature difference between mountainous and plain areas, with considerable annual and daily temperature variations. The precipitation pattern during the period of 1980–2019 did not exhibit a clear trend, with notable mutation points observed in 1983, 2004, 2009, and 2016. The distribution of precipitation shows higher levels in the south and lower levels in the north, with greater amounts observed in the southwest and reduced amounts in the northeast. However, these findings may be attributed to the 40-year time series selected for this study, causing slight variations in the results. Meanwhile, the cumulative sunshine hours, on the other hand, displayed an oscillating decline, progressively decreasing from north to south. Henan Province’s climate is classified as a warm–temperate to subtropical, humid–semi-humid monsoon climate, characterized by a significant continental influence [47,48,49,50]. This climate classification results in distinct seasons in Henan Province, with concurrent rainfall and warmth, which is favorable for agricultural development. However, the intricate and varied climate conditions, coupled with the frequent occurrence of meteorological disasters, pose challenges to local livelihoods and production [51,52,53,54].

### 4.2. Impact of Climate Change on the Planting Area of Winter Wheat

With urbanization and the general degradation of agricultural land, the reduction in the amount of arable agricultural land for cultivation is putting additional pressure on the global food supply (IPCC 2019). Therefore, more and more studies have been conducted to evaluate the impact of climate change on food security from the perspective of crop yield per unit area and crop planting area [55,56]. Global warming has extended the cultivation limits of crops to higher altitudes and latitudes [57]; this will certainly lead to changes in crop planting area. This study delves into the spatial distribution of winter wheat in Henan Province between 2000 and 2019, utilizing precise extraction techniques [58,59,60,61]. The analysis revealed a stabilizing trend in the overall cultivation structure of winter wheat during this period. Notably, global climate warming emerged as a pivotal factor shaping the suitable planting areas for winter wheat, where increasing temperatures enabled previously unsuitable regions to support winter wheat growth, thereby expanding the total planting area. In particular, this study observed a marked increase in the frequency of winter wheat cultivation in specific regions such as northwest Luoyang, north Pingdingshan, and northeast Zhumadian. Conversely, a decrease is noted in parts of Kaifeng and Zhengzhou. The accumulated temperature during the winter wheat growth period has been found to be positively correlated with the planting area of winter wheat, whereas the number of sunshine hours exerts an inhibitory effect. In ranking the importance of meteorological factors during the winter wheat growth period, the study established a hierarchy of *T* > *P* > *h*. Moreover, the escalation of extreme weather events, including droughts and floods, poses a significant threat to winter wheat growth, potentially leading to reduced production or even crop failure [62,63,64]. Furthermore, changes in winter wheat acreage is attributed to a complex interplay of natural, social, and economic factors, of which climate change was just one facet. This multifaceted analysis provides a comprehensive understanding of the dynamics shaping winter wheat cultivation in Henan Province [65].

### 4.3. Impact of Climate Change on the Yield of Winter Wheat

Climatic factors exert diverse impacts on winter wheat yield during the reproductive stage, leading to fluctuations that are dependent on prevailing weather conditions [66]. The above analysis shows that there is a positive correlation between accumulated temperature during the winter wheat growth period and its yield. Specifically, within a certain range, an increase in temperature and precipitation during this critical period is beneficial for the enhancement of winter wheat yield. Conversely, the duration of sunshine during the winter wheat growth period exhibits a moderate inhibitory effect on winter wheat yield. In Henan Province, meteorological factors during the winter wheat growth period were ranked in the following order of significance for winter wheat yield: *T* > *P* > *h*. Temperature is identified as the primary factor influencing the growth and development of winter wheat. Optimal temperature conditions are conducive to the various growth processes of winter wheat, including overwintering, rejuvenation, nodulation, and tillering. However, fluctuations in minimum temperature could potentially threaten the growth of winter wheat. Extreme weather events, i.e., sudden spring cold inversions, can result in frost damage and a subsequent yield reduction [67,68]. Furthermore, adequate levels of precipitation are essential for facilitating the necessary moisture conditions to facilitate the growth and development of winter wheat. However, both excessive and deficient precipitation can adversely affect winter wheat yield. Specifically, excessive precipitation might result in waterlogging, which can impede the respiration and nutrient absorption of the wheat root system. Conversely, insufficient precipitation could induce drought conditions, limiting photosynthesis and the accumulation of dry matter in wheat plants [69,70]. Moreover, the level of light exposure also plays a crucial role in determining the productivity of winter wheat. Optimal light availability not only enhances the photosynthetic process in wheat but also both yield and quality. Conversely, inadequate light levels can hinder wheat photosynthesis, subsequently affecting the accumulation of dry matter and ultimately influencing yield formation [18,71]. Therefore, a balanced combination of temperature, precipitation, and light exposure is essential for ensuring the optimal growth and development of winter wheat. The above studies have shown the correlation between meteorology-driven factors and yields, but the results of these studies have differed, due to the variety, scale, and geographical location, etc. [46,72]. The identification of climate change is crucial for formulating effective adaptation measures in Henan Province, the main producing area of winter wheat. It can be inferred that determining the meteorology-driven effects on crop yields in Henan Province, a major winter wheat producing area, is crucial for formulating effective regional adaptation measures to adapt to future climate challenges.

## 5. Conclusions

With global warming, precipitation, accumulated temperature, and sunshine duration, as typical agrometeorological driving factors, have also undergone significant changes in Henan Province, which will inevitably lead to changes in crop planting distribution pattern. In this study, GLASS LAI data were extracted from remote sensing images to obtain the information of winter wheat planting area, and the spatial distribution of winter wheat in Henan has been precisely delineated, revealing consistent patterns across years, and the primary cultivation areas are concentrated in the central, eastern, and northern regions. The distribution of winter wheat planting has remained relatively stable over the observation period, with a general increase in planting area. In addition, planting area is positively correlated with accumulated temperature during the reproductive phase. Conversely, it is negatively correlated with sunshine duration during this period. This study comprehensively examined the specific impact of climatic-factor changes on winter wheat planting area and yield. The results showed that winter wheat yield in Henan Province exhibited an overall upward trajectory, exhibiting a positive correlation with accumulated temperature during the reproductive phase. Conversely, yield was negatively correlated with sunshine duration during this critical phase. Furthermore, annual yield variations displayed a negative correlation with precipitation during the entire growth period of winter wheat. Notably, the results also indicate a strong negative relationship between yield variability and precipitation levels. This study enhances the understanding of meteorology-driven effects on planting distribution patterns and crop production but also provides a reference for future agricultural practices and climate adaptation strategies.

## Figures and Tables

**Table 1:** Correlation coefficients between meteorological factors and the planting area of winter wheat.

Meteorological Factor	Planting Area		Annual Difference in Planting Area		Yield		Annual Difference in Yield	
	Pearson Correlation	Significance *p*-Value	Pearson Correlation	Significance *p*-Value	Pearson Correlation	Significance *p*-Value	Pearson Correlation	Significance *p*-Value
Accumulated temperature	0.685 **	0.000	−0.188	0.246	0.765 **	0.000	0.087	0.593
Precipitation	0.083	0.609	0.030	0.855	0.005	0.977	−0.393 *	0.012
Sunshine hours	−0.637 **	0.000	−0.118	0.470	−0.614 **	0.000	0.126	0.437

*, significant at *p* < 0.05; **, significant at *p* < 0.01.

## Data Availability

Data are contained within the article.
